# 1056. Safety and Tolerability of Dalbavancin in Vancomycin Allergic Patients – A Case Series

**DOI:** 10.1093/ofid/ofab466.1250

**Published:** 2021-12-04

**Authors:** Kennedy J Freeman, Kerry O Cleveland, Christopher M Bland, Bruce M Jones

**Affiliations:** 1 University of South Carolina College of Pharmacy, Columbia, South Carolina; 2 University of Tennessee Health Science Center, Memphis, Tennessee; 3 University of Georgia College of Pharmacy, Savannah, GA; 4 St. Joseph’s/Candler Health System, Savannah, Georgia

## Abstract

**Background:**

VVancomycin and dalbavancin, both in the glycopeptide class of antibiotics, are used in the treatment of Gram-positive infections, including methicillin-resistant *Staphylococcus aureus*. Antibiotics in this class contain a heptapeptide core that has potential for cross-sensitivity. Due to this risk, dalbavancin carries a warning in the package insert for use in patients with a glycopeptide allergy. Dalbavancin, a semi-synthetic derivative of vancomycin, has lipophilic side chains which reduce the risk of cross-sensitivity to vancomycin. This case series evaluated patients with a listed vancomycin allergy in their electronic health record who received dalbavancin as an outpatient infusion.

**Methods:**

This study was a non-randomized, retrospective chart review of adult patients who had a documented vancomycin allergy and received dalbavancin between February 2016 and February 2021 for any indication in the outpatient setting. The primary objective was to evaluate dalbavancin tolerability in patients allergic to vancomycin. Patient characteristics and the specifics of dalbavancin infusion – dose, volume, infusion rate, intravenous line access, and receipt of premedication before infusion – were collected on each patient.

**Results:**

559 unique patients received dalbavancin over the time frame. Of these, ten had a documented, subjective vancomycin allergy. Patient-reported allergic reactions were rash (4), hives (3), anaphylaxis (2), red man syndrome (2), renal failure (2), and general malaise (1). Six patients had at least 1 additional subjective drug allergy. The various infections treated included cellulitis/abscess (8), osteomyelitis (1), and bacteremia (2). Most patients received 1500mg (2 received 1125mg) of dalbavancin in 300-500mL of dextrose 5% in water infused at either 600 or 1000mL/hr via a peripheral (6) or central (4) intravenous line. All patients tolerated the infusion with no adverse events reported and no receipt of premedication before administration.

**Conclusion:**

Dalbavancin may be a reasonable treatment option in vancomycin allergic patients, despite possible cross-sensitivity. Further investigation into cross-sensitivity between vancomycin, dalbavancin, and other glycopeptide class agents is warranted.

**Disclosures:**

**Kerry O. Cleveland, M.D.**, **AbbVie** (Speaker’s Bureau)**Merck** (Speaker’s Bureau)**Pfizer** (Speaker’s Bureau) **Bruce M. Jones, PharmD, BCPS**, **Abbvie** (Consultant, Advisor or Review Panel member, Speaker’s Bureau)**La Jolla** (Speaker’s Bureau)**Melinta** (Consultant)**Merck** (Consultant)**Paratek** (Consultant, Speaker’s Bureau)

